# A deep learning-based whole-body solution for PET/MRI attenuation correction

**DOI:** 10.1186/s40658-022-00486-8

**Published:** 2022-08-17

**Authors:** Sahar Ahangari, Anders Beck Olin, Marianne Kinggård Federspiel, Bjoern Jakoby, Thomas Lund Andersen, Adam Espe Hansen, Barbara Malene Fischer, Flemming Littrup Andersen

**Affiliations:** 1 Department of Clinical Physiology, Nuclear Medicine, and PET, Rigshospitalet, Copenhagen, Denmark; 2 Siemens Healthcare GmbH, Erlangen, Germany; 3 Department of Clinical Medicine, University of Copenhagen, Copenhagen, Denmark; 4 Department of Diagnostic Radiology, Rigshospitalet, Copenhagen, Denmark

**Keywords:** PET/MRI, MR-AC, Attenuation correction, Deep learning, Whole body

## Abstract

**Background:**

Deep convolutional neural networks have demonstrated robust and reliable PET attenuation correction (AC) as an alternative to conventional AC methods in integrated PET/MRI systems. However, its whole-body implementation is still challenging due to anatomical variations and the limited MRI field of view. The aim of this study is to investigate a deep learning (DL) method to generate voxel-based synthetic CT (sCT) from Dixon MRI and use it as a whole-body solution for PET AC in a PET/MRI system.

**Materials and methods:**

Fifteen patients underwent PET/CT followed by PET/MRI with whole-body coverage from skull to feet. We performed MRI truncation correction and employed co-registered MRI and CT images for training and leave-one-out cross-validation. The network was pretrained with region-specific images. The accuracy of the AC maps and reconstructed PET images were assessed by performing a voxel-wise analysis and calculating the quantification error in SUV obtained using DL-based sCT (PET_sCT_) and a vendor-provided atlas-based method (PET_Atlas_), with the CT-based reconstruction (PET_CT_) serving as the reference. In addition, region-specific analysis was performed to compare the performances of the methods in brain, lung, liver, spine, pelvic bone, and aorta.

**Results:**

Our DL-based method resulted in better estimates of AC maps with a mean absolute error of 62 HU, compared to 109 HU for the atlas-based method. We found an excellent voxel-by-voxel correlation between PET_CT_ and PET_sCT_ (*R*^2^ = 0.98). The absolute percentage difference in PET quantification for the entire image was 6.1% for PET_sCT_ and 11.2% for PET_Atlas_. The regional analysis showed that the average errors and the variability for PET_sCT_ were lower than PET_Atlas_ in all regions. The largest errors were observed in the lung, while the smallest biases were observed in the brain and liver.

**Conclusions:**

Experimental results demonstrated that a DL approach for whole-body PET AC in PET/MRI is feasible and allows for more accurate results compared with conventional methods. Further evaluation using a larger training cohort is required for more accurate and robust performance.

**Supplementary Information:**

The online version contains supplementary material available at 10.1186/s40658-022-00486-8.

## Background

Combined PET/MRI has gradually gained ground in routine clinical practice [1, 2]. The feasibility and utility of PET/MRI for evaluating a single body region have been highlighted in several recent reports [3, 4]. Its whole-body application has also gained attention lately as a potentially effective tool for the evaluation of oncologic diseases and detection of malignancies in various organs [5]. However, one of the major limitations of the PET/MRI has been the lack of a reliable attenuation correction (AC), which is required for accurate quantification of the radiotracer distribution. Several studies have developed innovative techniques for calculating accurate MR-based AC maps that have been shown to improve the quantitative accuracy of PET/MRI on a regional level [6–9]. However, a demonstration of whole-body implementation, covering skull to feet, is still lacking.

To convert MRI intensities to attenuation coefficients, there is no unique global mapping technique. Commercially available MR-based AC methods are based on a quickly acquired Dixon MRI sequence [10], combined with the segmentation of different tissue classes and assignment of proper linear attenuation coefficients (LACs) to each tissue class [11]. The segmentation can be combined with ﻿registration to atlas templates providing constant LACs for bone [12, 13]. However, such methods are not patient-specific and inaccurate registration between images and bone models could increase the risk of errors in PET quantification. Alternative approaches proposed the acquisition of ﻿ultrashort echo time (UTE) [14, 15] or zero echo time (ZTE) [16, 17] sequences to obtain a patient-specific signal from the bone. The performance of these methods is limited by issues like high level of noise or long acquisition time, which is not suitable for breath-hold imaging.

Moreover, a major limitation of conventional MR-based AC methods has been truncation due to the limited transaxial field of view (FOV) of MRI compared to PET [18]. Different approaches have been proposed to estimate the missing part of the anatomy from ﻿non-attenuation-corrected (NAC) PET [19] or using the maximum-likelihood reconstruction of activity and attenuation (MLAA) algorithm [20]. Alternatively, a fully MR-based approach with B0 homogenization using gradient enhancement (HUGE) sequence was proposed to compensate for the B0 inhomogeneities at the periphery of an increased MRI FOV [21]. The method showed significantly improved PET quantification but replaced the truncated anatomy with a single LAC value.

Recently, artificial intelligence algorithms based on deep learning (DL) convolutional neural networks (CNN), and generative adversarial network (GAN) have demonstrated remarkable potential as alternatives to conventional AC methods [22–25]. This approach can be time effective and robust to individual patient variations. Many DL-based studies are focused on cross-domain image transition to derive synthetic CT (sCT) images directly from MR images [26, 27]. This approach was initially developed for brain imaging due to the low geometric variance across different imaging modalities [26] but has proven robust and accurate, especially using a ﻿large training cohort [28]. Apart from the brain, ﻿several studies have been conducted to explore DL-based AC for other regions including head and neck [29], and pelvis [30, 31]. While these studies offer important contributions to improving PET AC in specific anatomical regions, the limited FOV hampers clinical adoption for whole-body PET/MRI. A few studies attempted to expand their method for AC of whole-body PET/MRI imaging [32, 33], ﻿but their results were not anatomically accurate across the entire body, possibly due to the challenges of producing well-aligned reference data throughout the body. To overcome this issue, DL-based methods have been proposed to generate sCT from NAC PET images [34] or directly generate AC PET from emission data [35]. However, NAC PET images do not provide explicit information about photon attenuation, but rely on information from the PET tracer of choice. Therefore, models generating sCT from NAC PET become tracer dependent and might work best for non-specific tracers like FDG that relay information from all parts of the body.

With this study, we aim to infer voxel-based sCT suitable for whole-body PET/MRI, covering skull to feet, and using standard Dixon MR images routinely acquired for AC purposes. The method will be based on paired whole-body Dixon MRI and CT data and a deep learning method and will be evaluated with reference to a CT-based PET reconstruction.

## Materials and methods

### Patients

The data in this study were acquired in two groups. First, a validation cohort of fifteen patients diagnosed with malignant melanoma who underwent whole-body acquisition. The imaging consists of a clinically indicated ^18^F-FDG-PET/CT examination and a subsequent whole-body PET/MRI acquisition. No additional radiotracer was injected for the PET/MRI acquisition. The second group was employed for model development and consisted of 11 patients scanned over head and neck (2 bed positions) and 20 patients scanned over the thorax and pelvis (2 bed positions). The imaging protocols and detailed information regarding these patients are described in previous studies [9, 29].

Written informed consent was obtained from all patients before the examination.

### Image acquisition

#### CT data

Each whole-body ^18^F-FDG-PET/CT examination (Biograph mCT, Siemens Healthineers) was performed with arms positioned alongside the body. Approximately 3 MBq/kg ^18^F-FDG was injected intravenously about 60 min prior to image acquisition. Standardized CT examination protocols included a weight-adapted 90–120 ml intravenous CT contrast agent, as part of the clinical routine. CT imaging was performed with a tube voltage of 120 kV, ﻿0.98 × 0.98 × 2 mm^3^ voxels, and a reference mAs of 240 using CARE Dose 4D. Data were acquired from the vertex of the skull to the toes using continuous table motion acquisition, except for two patients who were scanned from the skull to mid-thigh.

#### ^*18*^*F-FDG-PET/MRI data*

All PET/MRI examinations were performed on a 3 T PET/MRI whole-body hybrid imaging system (Biograph mMR, Siemens Healthineers) covering the same anatomy as in PET/CT. PET data were acquired in list mode over seven to eight bed positions with an acquisition time of 3 min per bed position.

For MR-based AC, the standard transaxial two-point Dixon, three-dimensional (3D), volume-interpolated, T1-weighted breath-hold MRI sequence (VIBE) was acquired utilizing a head/neck RF coil, a spine-array RF coil, and five flexible body array RF coil. MRI sequence was acquired with 3.85 ms repetition time (TR), 1.23 & 2.46 ms echo time (TE), and 10° flip angle for MR images with 384 × 312 pixel in-plane dimension. It provided four sets of MR images including T1-weighted in- and opposed-phase, fat and water images, with a transaxial MRI FOV of 500 mm × 408 mm.

To prevent truncation of the peripheral body parts due to the limited transaxial MRI FOV, the HUGE method was applied [21]. The sequence was acquired in the left and right direction with 1610 ms repetition time (TR), 27 ms echo time (TE), 180° flip angle, resolution of 2.3 × 2.3 mm^2^, and 8 mm slice thickness.

### Data preprocessing

Images were preprocessed using Python (version 3.7) and MINC Toolkit (McConnell Brain Imaging Centre, Montreal).

#### Truncation correction

The composed Dixon in-phase and opposed-phase images were combined with images acquired with the HUGE technique to correct truncation. HUGE images from the left and right side of the body were resampled to match the resolution of the Dixon images using trilinear interpolation. The histogram normalization was performed twice using the inormalize tool (version 1.5.1 for OS X as part of MINC toolkit) to match the intensity with Dixon in-phase and opposed-phase. The resampled and normalized HUGE images were then used to replace voxels in the Dixon images where the arms were truncated. Dixon images were extended by 18 voxels on each side, for a total transaxial FOV of 576 mm × 374 mm.

#### CT to MRI registration

Whole-body co-registration for the validation cohort was challenging due to the different positioning of patients between the two scans. To secure accurate co-registering of paired whole-body CT and MRI images, Dixon in-phase images were cropped into sub-volumes and registration was performed independently for various anatomical sites. Sub-volumes were defined by joints with a high degree of freedom which led to anatomical regions such as head, neck, torso, pelvic, upper arm, lower arm, upper leg, lower leg, and feet. For each sub-volume, registration was performed in two steps. First, CT images were rigidly aligned to the corresponding Dixon in-phase images using a set of landmarks. The transformation file was used to initialize a deformable registration using the freely available registration package, NiftyReg [36] (Centre for Medical Image Computing, University College London). Sub-volumes were drawn with an overlap of two voxels in which the co-registered CT was averaged. Finally, co-registered sub-volumes were stitched together forming the whole-body co-registered CT volume. A thorough visual inspection was performed to validate each individual registration.

Registration between CT and MRI for regional data was less challenging due to similar positioning between the two scans. However, registration was performed for each bed position separately using the method described above.

### Network structure

The deep CNN with 3D U-Net architecture [37] used in this study was developed with convolutional encoder/decoder parts for generating sCT in LAC from MR images (as shown in Additional file 1: Fig. S1). The overall architecture of our model was a slight modification of the DeepDixon network presented by Ladefoged et al. [23]. Dixon in-phase and opposed-phase MRI were the inputs to the network and integrated by the first convolutional layer with 32 different kernels. Each of the encoder and decoder paths contains 3 × 3 × 3 kernels, followed by a batch normalization (BN) for faster convergence and a rectified linear unit (ReLU) activation function. At the end of each convolution operation, a similar convolutional layer with strides of 2 is attached for downsampling.

### Model training

The training of the model was performed using pairs of Dixon MRI and CT-based AC volumes. Prior to the training, images were resampled to the isotropic voxel size of 2 mm and normalized to zero mean and unit standard deviation. A binary mask was derived from Dixon in-phase images to clear the CT images from elements outside the body, before transforming the voxels into LACs at 511 keV. Subsequently, we extracted 3D patches from 288 × 192 × S with a stride of 4, where S refers to the number of slices and varies between patients depending on their length. The networks were implemented in TensorFlow (version 2.1.0). Our model used mean absolute error as the loss function, Adam optimizer with a learning rate of 5 × 10^–5^ trained for 1000 epochs with a batch size of 16 (random selection of patches). Computations were performed on two IBM Power9 each equipped with four Nvidia Tesla V100 GPUs and a Lenovo SR650_v2 with four Nvidia A40 GPUs. The networks used 3D MRI volumes as a 2-channel input consisting of ﻿16 full adjacent transaxial slices (288 × 192 × 16 slices) and output the corresponding slices of sCT in LAC (288 × 192 × 16 slices). First model was trained using the regional data containing thorax, pelvis, and head and neck regions with transfer from a pretrained model on 811 brain scans [23]. Subsequently, we created an updated model using the whole-body database with transfer learning from the first model. In this step, we used a leave-one-out cross-validation approach. To avoid artificially increased bias, CT slices with a metal artifact from pelvis and knee were not included in the training. For each whole-body dataset, the sCT volume was generated from the MRI volumes.

### Image reconstruction

PET images from raw data acquired on PET/MRI scanner were reconstructed offline (e7-tool, Siemens Healthineers) using 3D ordinary Poisson ordered-subset expectation maximization (3D OP-OSEM) algorithm with 3 iterations, 21 subsets in 344 × 344 image matrix, and a Gaussian filter with 4 mm full width at half maximum (FWHM). For each patient, PET images were reconstructed using three different attenuation maps: co-registered CT-based AC map serving as a standard of reference (PET_CT_), deep learning-derived sCT map (PET_sCT_), and vendor-provided atlas-based map (PET_Atlas_).

### Analysis

Data analysis involved resampling all reconstructed PET images to match the voxel size of the attenuation map.

#### sCT evaluation

The generated sCT images in LAC were converted back to HU and compared to CT on a voxel-wise basis.

For each patient, the accuracy of sCT relative to CT was compared by measuring the mean absolute error (MAE) within the body contour. The ability of the methods to correctly estimate bony tissues was evaluated using the Dice similarity coefficient that measures the overlap between the segmented bones on sCT and CT volumes. In both images, voxels with HU higher than 300 were classified as bone.

#### PET evaluation

For quantitative assessment, the intensity values in PET images were converted to standardized uptake value (SUV). Considering PET_CT_ as the ground truth, the performance of MR-based AC methods in quantifying radiotracer uptake in PET_sCT_ and PET_Atlas_ were compared for the entire body as well as specific regions including lung, liver, spinal cord, femoral head, iliac bone, and aorta. These regions were manually segmented on the reference CT. Quantitative assessment was compared using relative difference (Rel%) and absolute relative difference (Abs%) for all the voxels within the above-mentioned regions, using the following formula:

$${\text{Rel}}\% = \frac{{{\text{PET}}_{X} - {\text{PET}}_{{{\text{CT}}}} }}{{{\text{PET}}_{{{\text{CT}}}} }} \times 100\%$$

$${\text{Abs}}\% = \frac{{\left| {{\text{PET}}_{X} - {\text{PET}}_{{{\text{CT}}}} } \right|}}{{{\text{PET}}_{{{\text{CT}}}} }} \times 100\%$$

For a fair comparison, PET slices with a metal artifact in CT-based AC map were ignored in the voxel-wise calculation of relative differences. The mean SUV (SUV_mean_) was calculated for the segmented regions.

Moreover, all the voxels within the specified regions were pooled over all subjects and the accuracy of the two MR-based AC maps on PET quantification was compared to PET_CT_ in a joint histogram.

## Results

Among the validation cohort of fifteen, one patient was suffering from severe scoliosis. Two patients had hip and knee implants, where the slices with artifact were excluded from training. In addition, one patient has scanned arms up in PET/CT, and therefore slices with arms were excluded from training.

### Accuracy of sCT

**Fig. 1:**
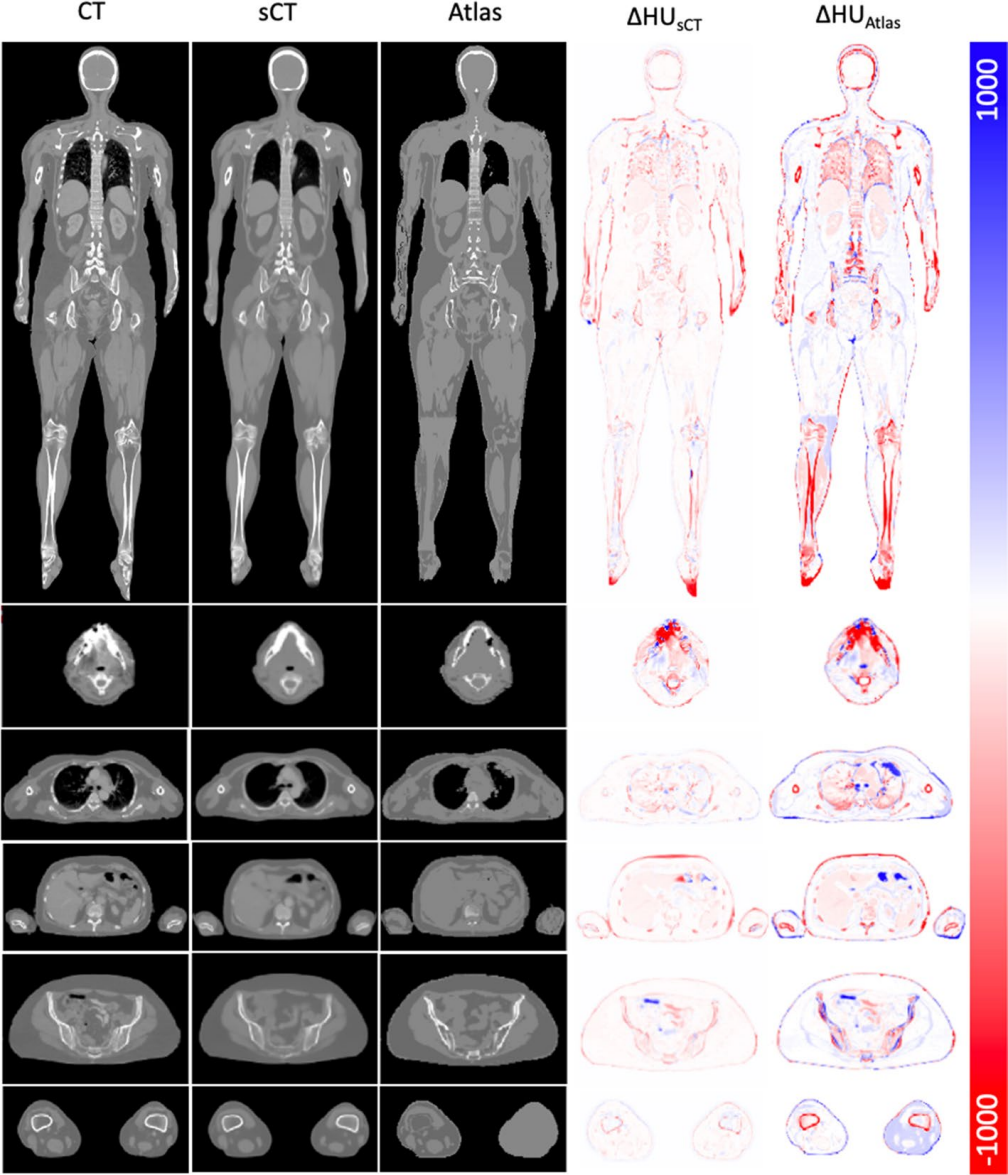
Whole-body coronal images of a representative patient as well as transaxial views of various intersections. Presented from left to right: reference CT, deep learning-derived sCT, vendor-provided atlas-based map, the HU difference between CT and sCT, and the HU difference between CT and atlas

Figure 1 illustrates the comparison between MR-based AC maps and the reference CT of a representative patient. The CT image of some patients showed streaking artifacts due to the dental implants. However, the CNN could generate sCT without these artifacts as exemplified in Fig. 1. The sCT appeared to be very similar to the CT except for some discrepancies in the bony structures and location of air cavities. The HU difference of the sCT compared to CT was smaller than vendor-provided atlas-based map with MAE within the body contours of 62 ± 110 and 109 ± 202, respectively. However, there is a slight underestimation of the sCT HU value as illustrated by the overall redness in the HU difference map in Fig. 1. The mean Dice coefficients for bone in sCT and atlas-based maps were 0.68 and 0.34, respectively.

### Effect on PET AC

**Fig. 2:**
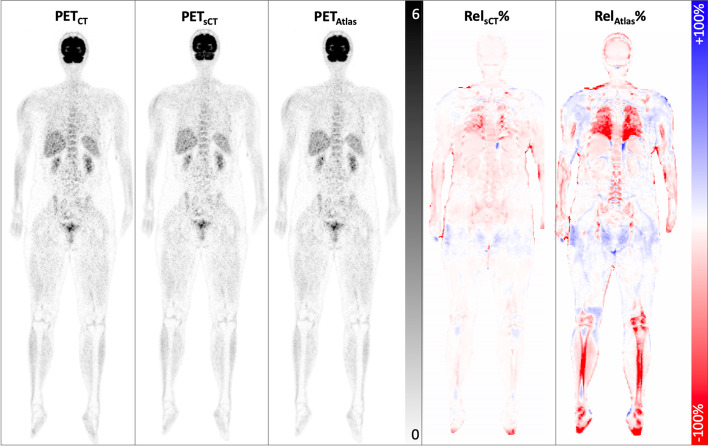
Representative images showing reference PET_CT_, PET_sCT_, and PET_Atlas_ images as well as the corresponding relative difference map of PET_sCT_ and PET_Atlas_ as compared to the reference PET_CT_

**Fig. 3:**
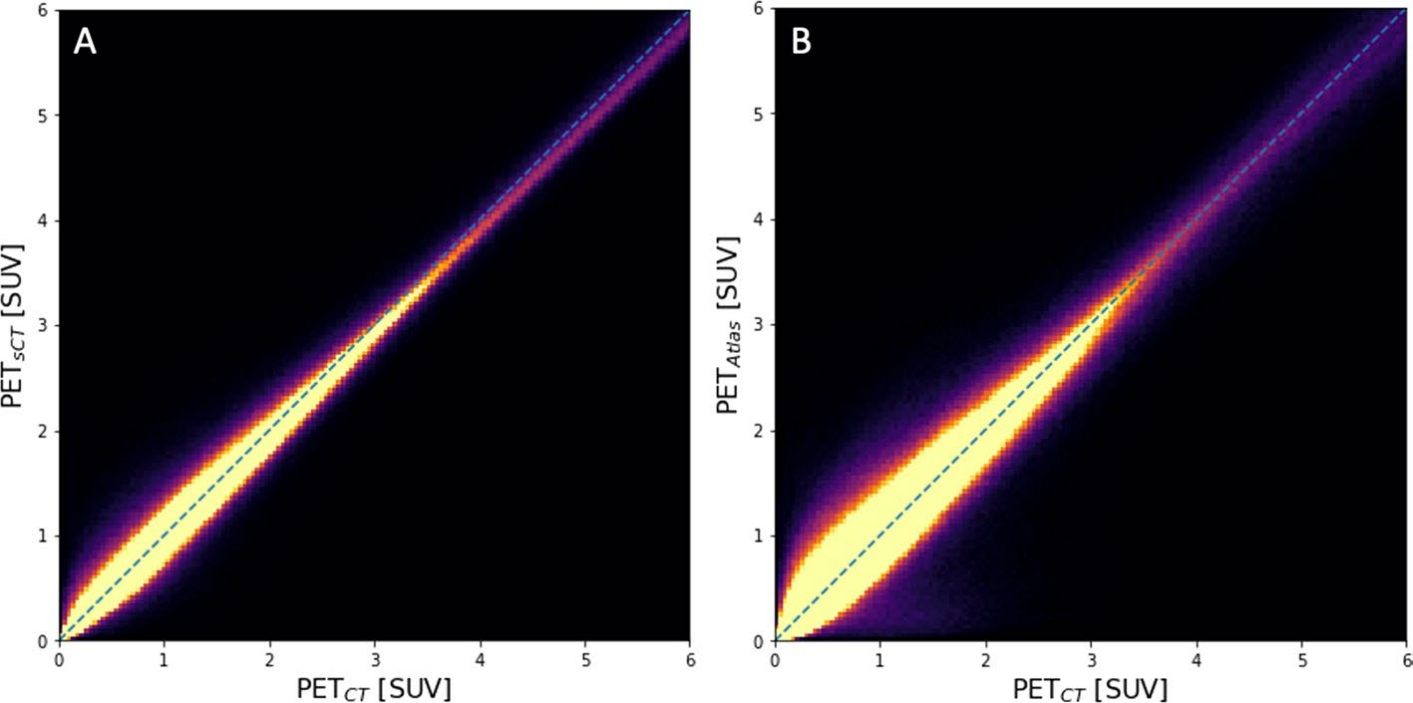
Joint histograms for **A** PET_CT_ and PET_sCT_, and **B** PET_CT_ and PET_Atlas_ of voxels within the body contour as well as lung, bone, and liver mask

The PET images reconstructed with different AC maps, PET_CT_, PET_sCT_, and PET_Atlas_, along with the percentage difference maps, are shown in Fig. 2. According to the difference maps, the DL-based method exhibited an overall superior performance to the vendor-provided method. PET_Atlas_ demonstrated the effect of errors in bone registration and also the absence of bone information in some regions, where the activity around the bony regions is underestimated. A Similar pattern can be observed for PET_sCT_, but with a considerably lower deviation. To further evaluate the performance of different AC maps in the entire body as well as at a regional level, joint histograms are shown in Fig. 3. The SUV value of PET_CT_ and PET_sCT_ (*R*^2^ = 0.98) is distributed closer to the equality line as compared to PET_CT_ and PET_Atlas_ (*R*^2^ = 0.96).

**Fig. 4:**
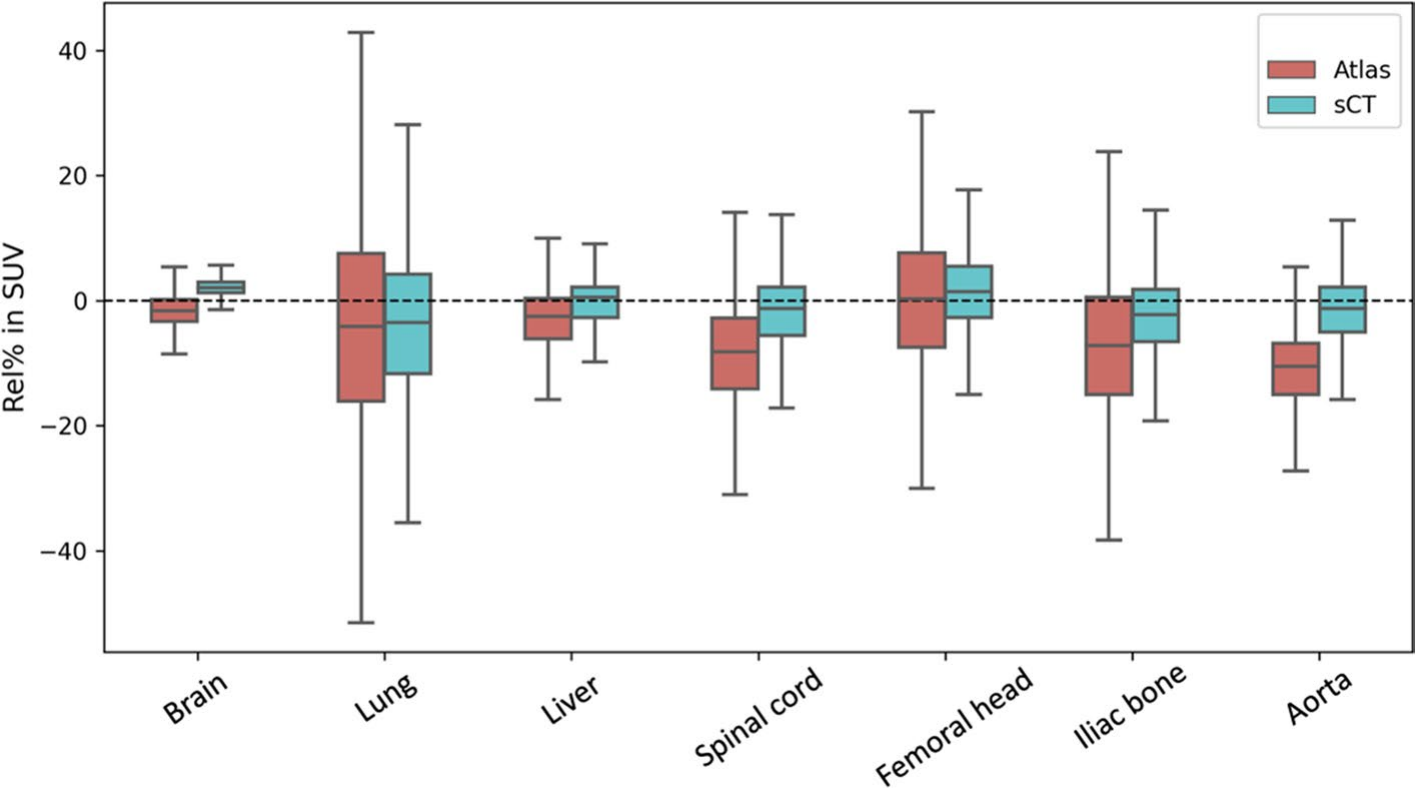
Percentage difference in PET SUV_mean_ of regions averaged across all patients for PET_Atlas_ (red) and PET_sCT_ (blue) compared with PET_CT_

PET_sCT_ produced an average Abs% of 6.1% ± 11.9% and an average Rel% of 1.5% ± 14.1% in SUV values for all the voxels within the body contour across all patients, while these quantitative metrics for PET_Atlas_ were 11.8% ± 23.6% and − 0.5% ± 26.2% respectively. Table 1 provides an average Abs% and Rel% of SUV values in PET_sCT_ and PET_Atlas_ for different anatomical regions. The comparison between the two methods in different regions is more evident in Fig. 4. PET_sCT_ generally has lower errors than PET_Atlas_ within all regions of interest except the liver. For both methods, the PET uptake in lung is underestimated and has the highest variation compared to other regions, while the uptake in liver has the least variation. The standard deviations of SUV differences with respect to the CT AC are always lower for PET_sCT_ than PET_Atlas_.

**Table 1:** Statistics for PET quantification errors of the two AC methods: the relative difference (Rel%) and absolute relative difference (Abs%) are reported for all the voxels within the specified regions

	PET_sCT_	PET_Atlas_
*Rel%*		
Brain	2.1 ± 2.4	− 2.1 ± 3.2
Lung	− 4.9 ± 12.1	− 4.3 ± 20.3
Liver	− 0.5 ± 4.4	− 0.4 ± 5.1
Spinal cord	− 2.4 ± 6.4	− 7.9 ± 9.7
Femoral head	0.5 ± 6.4	0.8 ± 12.1
Iliac bone	− 4.0 ± 6.5	− 7.7 ± 12.4
Aorta	− 0.8 ± 4.8	− 10.9 ± 7.2
*Abs%*		
Brain	1.9 ± 2.3	3.2 ± 2.3
Lung	9.5 ± 10.5	15.4 ± 13.9
Liver	3.4 ± 2.9	4.2 ± 2.7
Spinal cord	4.9 ± 4.7	9.8 ± 7.6
Femoral head	4.9 ± 4.1	9.3 ± 7.8
Iliac bone	5.8 ± 5.0	11.6 ± 8.9
Aorta	3.8 ± 2.9	11.2 ± 6.7

**Fig. 5:**
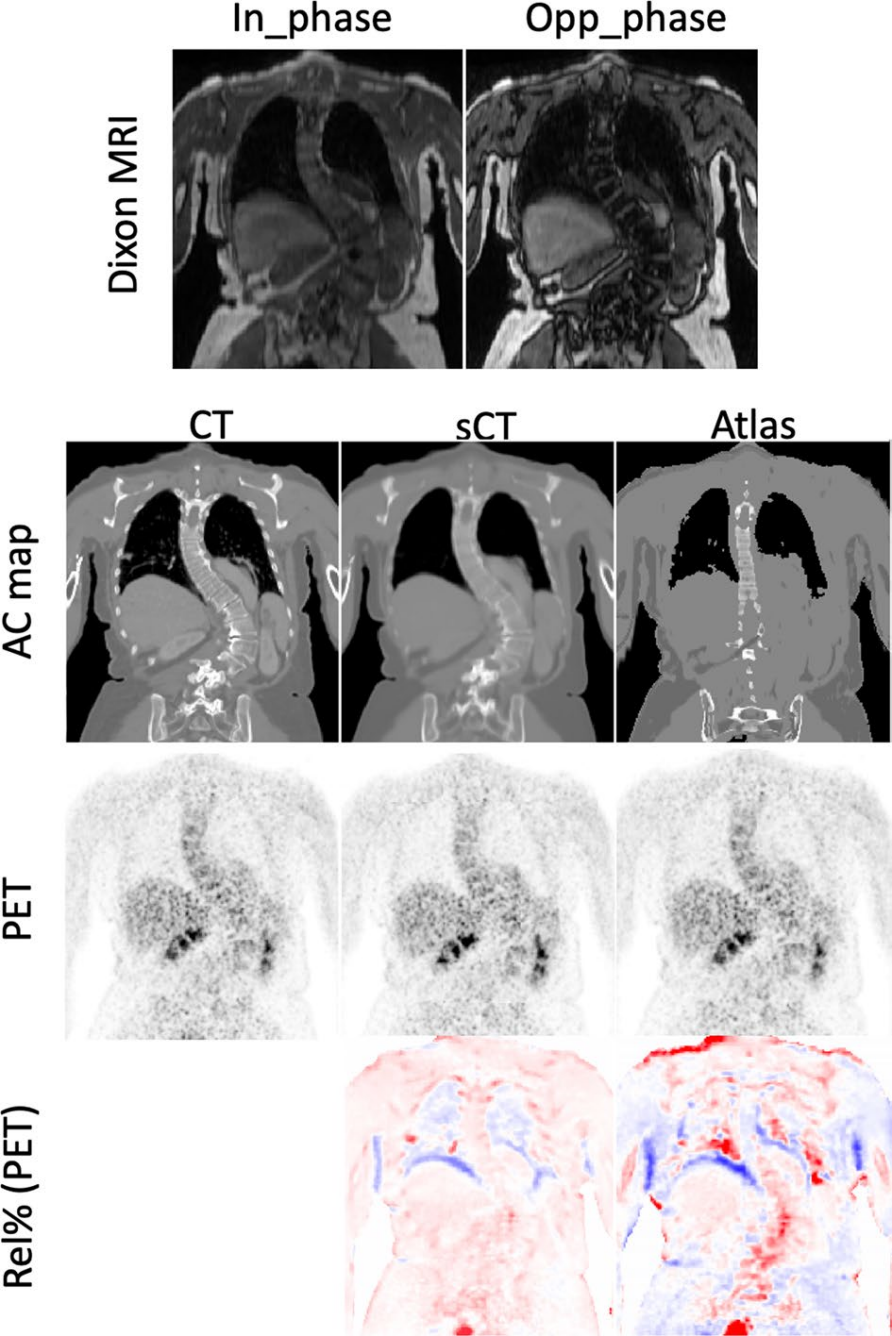
A clinical case with severe scoliosis. Dixon MRI images (first row); Reference CT, sCT, and atlas-based AC maps (second row); corresponding PET image (third row); percentage difference map (last row)

Apart from the overall performance of both methods, we wished to investigate how our DL-based AC method performs on a patient with abnormalities. Figure 5 depicts AC maps and PET images of the patient with scoliosis presented with the percentage difference maps. The vendor-provided atlas-based method was not able to adapt to the sideways curvature of the spine, which lead to significant quantification error around the spine. The DL-based method, however, showed promising results in generating an sCT volume that accurately reflected the abnormal anatomy and consequently minimal SUV bias.

## Discussion

Our study explored the feasibility of a DL approach for more accurate and robust whole-body attenuation correction in PET/MRI systems. The application of the conventional atlas-based method for whole-body PET/MRI is challenging because of its limitation in accurate bone estimation as well as arms truncation and anatomical variations in the chest and abdomen. Here we performed truncation correction of Dixon images and used them as the training inputs to generate DL-based sCT which proved an effective tool for PET AC and outperformed the ﻿current clinical approach.

The DL method employed in this study has previously been explored for different regions [23, 30]. However, the novelty of this study is the development of a model addressing whole-body PET/MRI AC. Qualitative analyses of sCT provided results similar to reference CT and indicated a better estimation of patient-specific AC map, particularly in lung and bone. Our method showed a better estimation of AC map compared to the atlas-based method. The largest inaccuracies of the sCT were observed at the bone boundaries in pelvis and spine, possibly due to the slightly blurred appearance of the sCT and inaccuracies in MRI to CT registration. The atlas-based method shows relatively large discrepancies in the bony structures mostly due to inaccurate atlas registration and missing bones in some regions. Moreover, sCT was exposed to considerable variations in air pocket location in the abdomen due to different air configuration between MRI and CT acquisitions. However, sCT is expected to align well with MRI images, while the atlas-based method cannot handle air pockets in AC maps.

A quantitative PET evaluation showed an average underestimation of 1.5% using the DL-based method, compared to the CT-based reference. Notably, the absolute error was significantly lower for PET_sCT_ (6.1%) than for PET_Atlas_ (11.8%) signifying a more accurate estimation of tracer uptake as also shown by smaller standard deviations of errors. Joint histogram plots indicate that the DL-based AC method produces PET images that are more similar to the reference than the vendor-provided atlas-based method. The narrow distribution for PET_sCT_ suggests it is more accurate than the PET_Atlas_.

PET_sCT_ demonstrated the lowest errors in comparison with PET_Atlas_ in all regions; however, the largest bias was observed in lung and the smallest biases occurred in brain and liver. The atlas-based method allocated a predefined attenuation coefficient to the entire lung, which led to the overall underestimation of PET tracer uptake, whereas sCT images displayed a continuous density signal in this region. An inaccurate estimation of lung tissue LAC was observed in sCT, but its impact on PET quantification was less severe. Also in earlier PET/MRI AC publications, the lung demonstrated a substantially larger error compared with other body regions [12, 37]. Regions representing bone also showed significant errors in PET_sCT_, especially iliac bone in pelvis due to the high density of bone in this region.

A previous evaluation of the conventional method for whole-body PET/MRI AC was in line with our observations reporting an underestimation of − 4.9% ± 7.7% in bone [12]. Our DL model reduced the error from − 8.7 to − 2.3% in bone, excluding the lower legs where the atlas-based method does not include bone at all.

Some studies have utilized diagnostic MR images to generate AC maps with DL approaches [28, 38]. Despite the more accurate anatomical information in diagnostic images, it is not practical for whole-body scans, given the long scan time and different contrast of diagnostic MR images. Development of our DL method relies on a unique dataset of paired co-registered whole-body Dixon MRI and CT-based AC volumes for training, representing a variety of anatomical features. It has the strength of using a fast sequence which is less sensitive to anatomical variation and patient movement. Dixon MR images have been investigated for the pelvic region as training inputs alone [31] and in combination with ZTE images [30], also leading to a reduction in PET quantification errors.

The findings of previous studies involving DL-based AC mainly stem from a regional analysis of 18F-FDG PET. In our previous work [9], we demonstrated the feasibility of the proposed method using a different radiotracer, 68 Ga-RGD, in the pelvic region. In contrast, only limited research has been done on the whole-body application of DL-based techniques due to the ﻿limited availability of paired whole-body ﻿CT and MR images [33]. To overcome the challenge regarding whole-body registration between the two scans, methods using CycleGAN have been proposed for training on unpaired data and showed promising results for brain [39]. Another DL approach relied on NAC PET images for whole-body AC PET, which fails in complex anatomical regions due to the lack of structural information and has shown relatively high error in the lungs [35]. Recent studies relying on NAC PET images improved the accuracy of AC maps by simultaneous reconstruction of the activity in PET images using MLAA algorithm [40]. This method is not feasible without time-of-flight method and was limited with the activity distribution which resulted in a high level of noise on the AC map [41].

Moreover, the main concern about all MRI-based AC methods is their performance for cases with abnormalities. A recent study compared the performance of the atlas-based method with a DL-based approach in cases with severe body truncation, metal artifacts, abnormal anatomy, and lesions in the lungs [42]. They reported overall promising results for sCT and suggested more detailed studies addressing metal artifact and body truncation issues. In our model, the truncation artifact was significantly reduced compared to similar studies [43, 44]. It also outperformed the atlas-based method in a case with severe scoliosis, where the conventional method was unable to predict the sideway curve of the spine correctly. In some of the cases with dental implants, the extent of the artifact in MR images was localized to a greater extent than in CT as shown in Fig. 1, which made sCT a valuable source for predicting anatomical information in a corrupted region. Other studies, however, reported failures of the DL-based method in patients with dental artifacts [23].

This study had certain limitations. First, our training dataset was relatively small. Training data are a significant contributor to overall model performance [23]. However, transfer learning has been used to overcome small training group sizes to some extent. Additionally, due to the different bed shapes and patient positioning between the two scans, our training quality was prone to inaccuracies as a result of registration errors between MRI and CT. A relatively large mismatch was observed for the patient arms, due to the higher degree of freedom. It should also be mentioned that CT is not an ideal reference for training since CT cannot directly reflect the attenuation information for photons at 511 keV. It may be possible in the future to use a reproducible positioning system for whole-body imaging and a larger database containing the subjects with anatomical abnormalities to improve the robustness of the model. Different training and testing datasets obtained from different scanners also would be valuable to evaluate the clinical translation of our method.

## Conclusion

This study demonstrated the feasibility of a DL method to improve MR-based PET attenuation correction in whole-body PET/MRI, covering skull to feet. The sCT images, inferred from routinely acquired standard Dixon MR images, demonstrated only a small quantification error compared to a CT reference and better performance than the conventional vendor-provided atlas-based method. The sCT significantly improved PET AC in bone and lung proved and is likely to be a valuable tool for clinical implementation.

## Supplementary Information

**Additional file 1: Fig. S1.** CNN network in U-net architecture used in this study.

## Data Availability

﻿The datasets used and/or analyzed during the current study are available from the corresponding author on reasonable request and given approval from relevant regulatory authorities.

## Abbreviations

AC: Attenuation correction
DL: Deep learning
CNN: Convolutional neural networks
LAC: Linear attenuation coefficient
UTE: Ultrashort echo time
ZTE: Zero echo time
NAC: Non-attenuation-corrected
MLAA: Maximum likelihood estimation of activity and attenuation
HUGE: Homogenization using gradient enhancement
GAN: Generative adversarial network
sCT: Synthetic computed tomography
TR: Repetition time
TE: Echo time
FWHM: Full width at half maximum
MAE: Mean absolute error
SUV: Standardized uptake value

## References

[1] Mayerhoefer ME, Prosch H, Beer L, Tamandl D, Beyer T, Hoeller C (2020). PET/MRI versus PET/CT in oncology: a prospective single-center study of 330 examinations focusing on implications for patient management and cost considerations. Eur J Nucl Med Mol Imaging.

[2] Schwartz M, Gavane SC, Bou-Ayache J, Kolev V, Zakashansky K, Prasad-Hayes M (2018). Feasibility and feasibility and diagnostic performance of hybrid pet/mri compared with pet/ct for gynecological malignancies: a prospective pilot study. Abdom Radiol.

[3] Even AJG, De Ruysscher D, van Elmpt W (2016). The promise of multiparametric imaging in oncology: How do we move forward?. Eur J Nucl Med Mol Imaging.

[4] Ahangari S, Littrup Andersen F, Liv Hansen N, Jakobi Nøttrup T, Berthelsen AK, Folsted Kallehauge J (2022). Multi-parametric PET/MRI for enhanced tumor characterization of patients with cervical cancer. Eur J Hybrid Imaging.

[5] Martin O, Schaarschmidt BM, Kirchner J, Suntharalingam S, Grueneisen J, Demircioglu A (2020). PET/MRI versus PET/CT for whole-body staging: results from a single-center observational study on 1,003 sequential examinations. J Nucl Med.

[6] Mehranian A, Arabi H, Zaidi H (2016). Vision 20/20: magnetic resonance imaging-guided attenuation correction in PET/MRI: challenges, solutions, and opportunities. Med Phys.

[7] Wagenknecht G, Kaiser HJ, Mottaghy FM, Herzog H (2013). MRI for attenuation correction in PET: methods and challenges. Magn Reson Mater Physics, Biol Med.

[8] Lee JS (2021). A review of deep-learning-based approaches for attenuation correction in positron emission tomography. IEEE Trans Radiat Plasma Med Sci.

[9] Ahangari S, Hansen NL, Olin AB, Nøttrup TJ, Ryssel H, Berthelsen AK (2021). Toward PET/MRI as one-stop shop for radiotherapy planning in cervical cancer patients. Acta Oncol (Madr).

[10] Martinez-Moller A, Souvatzoglou M, Delso G, Bundschuh RA, Chefdotel C, Ziegler SI (2009). Tissue classification as a potential approach for attenuation correction in whole-body PET/MRI: evaluation with PET/CT data. J Nucl Med.

[11] Hofmann M, Bezrukov I, Mantlik F, Aschoff P, Steinke F, Beyer T (2011). MRI-based attenuation correction for whole-body PET/MRI: quantitative evaluation of segmentation- and atlas-based methods. J Nucl Med.

[12] Paulus DH, Quick HH, Geppert C, Fenchel M, Zhan Y, Hermosillo G (2015). Whole-body PET/MR imaging: quantitative evaluation of a novel model-based MR attenuation correction method including bone. J Nucl Med.

[13] Farjam R, Tyagi N, Deasy JO, Hunt MA (2019). Dosimetric evaluation of an atlas-based synthetic CT generation approach for MR-only radiotherapy of pelvis anatomy. J Appl Clin Med Phys.

[14] Keereman V, Fierens Y, Broux T, De Deene Y, Lonneux M, Vandenberghe S (2010). MRI-based attenuation correction for PET/MRI using ultrashort echo time sequences. J Nucl Med.

[15] Ladefoged CN, Benoit D, Law I, Holm S, Kjær A, Hjgaard L (2015). Region specific optimization of continuous linear attenuation coefficients based on UTE (RESOLUTE): application to PET/MR brain imaging. Phys Med Biol.

[16] Wiesinger F, Bylund M, Yang J, Kaushik S, Shanbhag D, Ahn S (2018). Zero TE-based pseudo-CT image conversion in the head and its application in PET/MR attenuation correction and MR-guided radiation therapy planning. Magn Reson Med.

[17] Leynes AP, Yang J, Shanbhag DD, Kaushik SS, Seo Y, Hope TA (2017). Hybrid ZTE/Dixon MR-based attenuation correction for quantitative uptake estimation of pelvic lesions in PET/MRI. Med Phys.

[18] Schramm G, Langner J, Hofheinz F, Petr J, Lougovski A, Beuthien-Baumann B (2013). Influence and compensation of truncation artifacts in MR-based attenuation correction in PET/MR. IEEE Trans Med Imaging.

[19] Delso G, Martinez-Möller A, Bundschuh RA, Nekolla SG, Ziegler SI (2010). The effect of limited MR field of view in MR/PET attenuation correction. Med Phys.

[20] Nuyts J, Bal G, Kehren F, Fenchel M, Michel C, Watson C (2013). Completion of a truncated attenuation image from the attenuated PET emission data. IEEE Trans Med Imaging.

[21] Blumhagen JO, Ladebeck R, Fenchel M, Scheffler K (2013). MR-based field-of-view extension in MR/PET: B0 homogenization using gradient enhancement (HUGE). Magn Reson Med.

[22] Lee JS (2020). A review of deep learning-based approaches for attenuation correction in positron emission tomography. IEEE Trans Radiat Plasma Med Sci.

[23] Ladefoged CN, Hansen AE, Henriksen OM, Bruun FJ, Eikenes L, Øen SK (2019). AI-driven attenuation correction for brain PET/MRI: clinical evaluation of a dementia cohort and importance of the training group size. Neuroimage.

[24] Nie D, Trullo R, Lian J, Wang L, Petitjean C, Ruan S (2018). Medical image synthesis with deep convolutional adversarial networks. IEEE Trans Biomed Eng.

[25] Arabi H, Zeng G, Zheng G, Zaidi H (2019). Novel adversarial semantic structure deep learning for MRI-guided attenuation correction in brain PET/MRI. Eur J Nucl Med Mol Imaging.

[26] Han X (2017). MR-based synthetic CT generation using a deep convolutional neural network method. Med Phys.

[27] Song X, Qian P, Zheng J, Jiang Y, Xia K, Traughber B (2020). mDixon-based synthetic CT generation via transfer and patch learning. Pattern Recognit Lett.

[28] Liu F, Jang H, Kijowski R, BradshawMcmillan TAB (2018). Deep learning Mr imaging-based attenuation correction for PeT, Mr imaging 1 TECHNICAL DEVELOPMENTS: Deep Learning MR Imaging-based Attenuation Correction for PET, MR Imaging Liu et al.. Radiol Radiol.

[29] Olin AB, Hansen AE, Rasmussen JH, Jakoby B, Berthelsen AK, Ladefoged CN (2022). Deep learning for Dixon MRI-based attenuation correction in PET/MRI of head and neck cancer patients. EJNMMI Phys.

[30] La P, Yang J, Wiesinger F, Kaushik SS, Shanbhag DD, Seo Y (2018). Zero-echo-time and dixon deep pseudo-CT (ZeDD CT): Direct generation of pseudo-CT images for Pelvic PET/MRI Attenuation Correction Using Deep Convolutional Neural Networks with Multiparametric MRI. J Nucl Med.

[31] Torrado-Carvajal A (2019). Dixon-vibe deep learning (divide) pseudo-CT synthesis for pelvis PET/MR attenuation correction. J Nucl Med.

[32] Ge Y, Xue Z, Cao T, Liao S. Unpaired whole-body MR to CT synthesis with correlation coefficient constrained adversarial learning. 2019;4.

[33] Schaefferkoetter J, Yan J, Moon S, Chan R, Ortega C, Metser U (2021). Deep learning for whole-body medical image generation. Eur J Nucl Med Mol Imaging.

[34] Dong X, Wang T, Lei Y, Higgins K, Liu T, Curran WJ (2019). Synthetic CT generation from non-attenuation corrected PET images for whole-body PET imaging. Phys Med Biol.

[35] Dong X, Lei Y, Wang T, Higgins K, Liu T, Curran WJ (2020). Deep learning-based attenuation correction in the absence of structural information for whole-body PET imaging. Phys Med Biol.

[36] Modat M, Ridgway GR, Taylor ZA, Lehmann M, Barnes J, Hawkes DJ (2010). Fast free-form deformation using graphics processing units. Comput Methods Programs Biomed.

[37] Lillington J, Brusaferri L, Kläser K, Shmueli K, Neji R, Hutton BF (2020). PET/MRI attenuation estimation in the lung: a review of past, present, and potential techniques. Med Phys.

[38] Bradshaw TJ, Zhao G, Jang H, Liu F, McMillan AB (2018). Feasibility of deep learning-based PET/MR attenuation correction in the pelvis using only diagnostic MR images. Tomogr (Ann Arbor, Mich).

[39] Gong K, Yang J, Larson PEZ, Behr SC, Hope TA, Seo Y (2021). MR-based attenuation correction for brain PET using 3-D cycle-consistent adversarial network. IEEE Trans Radiat Plasma Med Sci.

[40] Hwang D, Kim KY, Kang SK, Choi H, Seo S, Paeng JC, et al. Accurate attenuation correction for whole-body Ga-68-DOTATOC PET studies using deep learning. Soc Nuclear Med; 2019.

[41] Hwang D, Kang SK, Kim KY, Seo S, Paeng JC, Lee DS (2019). Generation of PET attenuation map for whole-body time-of-flight 18F-FDG PET/MRI using a deep neural network trained with simultaneously reconstructed activity and attenuation maps. J Nucl Med.

[42] Arabi H, Zaidi H (2022). MRI-guided attenuation correction in torso PET/MRI: Assessment of segmentation-, atlas-, and deep learning-based approaches in the presence of outliers. Magn Reson Med.

[43] Lindemann ME, Oehmigen M, Blumhagen JO, Gratz M, Quick HH (2017). MR-based truncation and attenuation correction in integrated PET/MR hybrid imaging using HUGE with continuous table motion. Med Phys.

[44] Grafe H, Lindemann ME, Ruhlmann V, Oehmigen M, Hirmas N, Umutlu L (2020). Evaluation of improved attenuation correction in whole-body PET/MR on patients with bone metastasis using various radiotracers. Eur J Nucl Med Mol Imaging.

