# Supplementary figures and images for: A deep learning-based whole-body solution for PET/MRI attenuation correction
Source: EJNMMI Phys. 2022 Aug 17;9:55. doi: 10.1186/s40658-022-00486-8 (PMC9385907; doi:10.1186/s40658-022-00486-8)

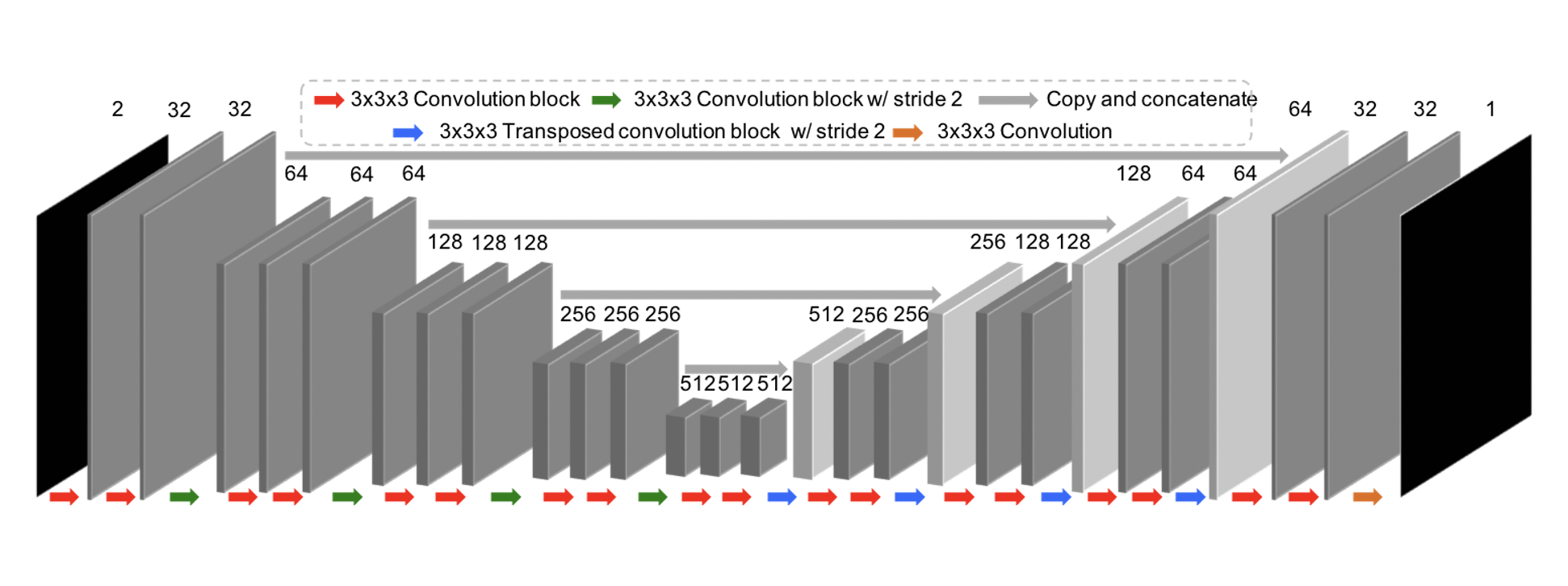

Supplement: Supplementary file 1 — Additional file 1: Fig. S1. CNN network in U-net architecture used in this study. [file 40658_2022_486_MOESM1_ESM.png]
